# Natural Spike Trains Trigger Short- and Long-Lasting Dynamics at Hippocampal Mossy Fiber Synapses in Rodents

**DOI:** 10.1371/journal.pone.0009961

**Published:** 2010-04-01

**Authors:** Anja Gundlfinger, Jörg Breustedt, David Sullivan, Dietmar Schmitz

**Affiliations:** 1 Neuroscience Research Center of the Charité, Universitätsmedizin Berlin, Berlin, Germany; 2 Bernstein Center for Computational Neuroscience, Humboldt Universität zu Berlin, Berlin, Germany; 3 Center for Molecular and Behavioral Neurobiology, Rutgers University, Newark, New Jersey, United States of America; Medical College of Georgia, United States of America

## Abstract

**Background:**

Synapses exhibit strikingly different forms of plasticity over a wide range of time scales, from milliseconds to hours. Studies on synaptic plasticity typically use constant-frequency stimulation to activate synapses, whereas *in vivo* activity of neurons is irregular.

**Methodology/Principal Findings:**

Using extracellular and whole-cell electrophysiological recordings, we have here studied the synaptic responses at hippocampal mossy fiber synapses *in vitro* to stimulus patterns obtained from *in vivo* recordings of place cell firing of dentate gyrus granule cells in behaving rodents. We find that synaptic strength is strongly modulated on short- and long-lasting time scales during the presentation of the natural stimulus trains.

**Conclusions/Significance:**

We conclude that dynamic short- and long-term synaptic plasticity at the hippocampal mossy fiber synapse plays a prominent role in normal synaptic function.

## Introduction

Synaptic plasticity changes the transmission efficacy at neuronal connections short- and longlastingly in an activity- and experience-dependent manner. Many characteristic features and important signaling cascades involved in different forms of plasticity have been elucidated by the use of acute slice preparations [1], [2]. In this context, the overwhelming majority of studies have used induction paradigms which consist of constant frequency stimulation patterns, like paired pulses, a tetanic stimulus or a theta burst protocol. A major drawback of all these induction paradigms is that they most likely do not resemble naturally occurring discharge patterns of single neurons *in vivo*.

A prominent trait of neuronal action potential discharges *in vivo* in the hippocampus is that specific neurons fire at an elevated rate when an animal traverses a certain position in space - the so-called place field of that neuron [3]. An extensive number of studies have elucidated general characteristics, mechanisms and function of such place-field activity in CA3 and CA1 pyramidal neurons of the hippocampus [4], [5]. Rather little, however, is known of place field related spiking activity in dentate gyrus granule cells [6], [7], as hippocampal granule cells are exceptionally hard to identify in long-term tetrode recordings due to their low discharge rate, small size and dense packing [8].

Such data are all the more interesting since the hippocampal mossy fiber synapse, here referring to the connection between granule cells and CA3 pyramidal cells, has received special interest for its peculiar short-term as well as long-term plasticity characteristics [9], [10]. The mossy fiber synapse serves as a model system for presynaptically induced and expressed long-term potentiation [11]. However, a limitation of the existing literature is that static and rather unphysiological stimulation paradigms typically were used to study plasticity and related signaling cascades at this synapse. We have recently provided a comprehensive description of mossy fiber synaptic short-term dynamics employing irregular stimulus trains [12], that were motivated by mean spike train statistics of dentate gyrus granule cells [13]. These were, however, still modelled spike trains and limited in the inter-spike interval range to values between 50 ms and 50 s, omitting high-frequency bursts of action potentials during place field crossings.

In this study, we have now investigated the effects of naturally occurring stimulus patterns on synaptic transmission at mossy fiber synapses in acute hippocampal slice preparations. For this purpose, we activated mossy fibers with stimulus patterns obtained from *in vivo* recordings of granule cell place field activity and found both large short-term facilitation and presynaptically induced and expressed long-term potentiation upon activation with place field associated spike trains.

## Materials and Methods

### Ethical guidelines

All animal experiments were performed according to national and institutional guidelines of the Charité - Universitätsmedizin Berlin (*in vitro* electrophysiology) and the Center for Molecular and Behavioral Neurobiology at Rutgers University (*in vivo* tetrode extracellular unit recordings).

### *In vivo* recordings and granule cell identification

LFP and unit activity was recorded during active waking behaviour and sleep from one adult male Long-Evans rat (500g), which was fed *ad libitum*. A 64-site silicon recording probe (8 shanks, 8 recording sites per shank, 200 µm lateral spacing between shanks, 20 µm vertical spacing between recording sites) was implanted into the dorsal right hippocampal CA1 region under isoflurane anesthesia. A bipolar electrode was implanted into the right angular bundle for perforant path stimulation. Ground and reference screws were implanted above the midline of the cerebellum. After implantation, the probe was lowered into the hippocampus by 37–150 µm at the end of each day until the evoked LFP response to perforant path stimulation reversed polarity from negative to positive. Anatomical locations of the silicon probe shanks were verified post-mortem via Nissl staining. Electrophysiological signals were acquired continuously at 32 kHz on a 128-channel, 16-bit Digital Lynx system (Neuralynx, Inc.). The rat was water deprived to encourage foraging for randomly sprinkled droplets of water in the open environment (1.95 m×1.20 m). The rat's position in the open environment was recorded via an overhead camera. Recorded signals were filtered below 800 Hz and thresholded for spike detection. Unit isolation was performed post-hoc using the KlustaKwik software package [14], followed by manual clustering using Klusters software [15]. An isolated unit was considered to be a putative granule cell if it (1) was recorded during a session in which the recording sites were located in the granule cell layer and (2) its spike autocorrelogram indicated spike bursts. Spike train 1 used in this study was derived as described above. Spike train 2 was taken from a previous publication (Henze *et al.*, 2002) and, in brief, consists of a short episode of granule cell firing recorded in a behaving mouse during a traversal of its respective place field.

### Brain slice preparation

Hippocampal slices were prepared from C57/Bl6 mice (P16–42) as previously described [16]. In brief, animals were anesthetized with isoflurane, decapitated and the brains removed. Tissue blocks containing the subicular area and hippocampus were mounted on a Vibratome (Leica VT1200S, Leica Microsystems, Wetzlar, Germany), in a chamber filled with ice-cold artificial cerebrospinal fluid (ACSF), containing (in mM): 87 NaCl; 75 sucrose; 25 NaHCO_3_, 2.5 KCl, 1 NaH_2_PO_4_, 0.5 CaCl_2,_ 7 MgSO_4_, 10 glucose, pH 7.4. Slices were cut at 300–400 µm thickness and heated to 35C° for 30 minutes. The slices were then cooled to room temperature and transferred to ACSF containing (in mM): 124 NaCl, 26 NaHCO_3_, 10 glucose, 3 KCl, 2.5 CaCl_2_, 1.3 MgSO_4_, 1.25 NaH_2_PO_4_. All ACSF was equilibrated with 95% O_2_ and 5% CO_2_. The slices were stored in a submerged chamber for 1-5 hours before being transferred to the recording chamber, where they were perfused with ACSF at a rate of 3-4 ml/min.

### Field potential and whole-cell recordings

Whole cell voltage-clamp and field excitatory postsynaptic potential (fEPSP) recordings were performed using a Multiclamp 700A (Axon Instruments, Foster City, CA, USA). Data were digitized (National Instruments BNC-2090) at 5–10 kHz and recorded and analyzed with custom-made software in IGOR Pro (WaveMetrics Inc., OR, USA). Patch electrodes (with electrode resistances ranging from 3 to 6 MΩ) were filled with (in mM): 135 K-gluconate, 20 KCl, 2 MgATP, 10 HEPES, 0.2 EGTA, 5 phosphocreatine, and adjusted to pH 7.3. Series resistances were 10.8±1.1 MΩ (, mean±stdev), and were continuously checked during the recordings and not allowed to vary more than 25% during the course of the experiment. fEPSP recordings were performed with low resistance patch pipettes filled with external solution placed in stratum lucidum. Mossy fibers were extracellularly stimulated with patch pipettes filled with external solution and placed in the granule cell layer or in the hilus region. Mossy fiber origin of recorded signals was routinely verified by frequency facilitation >400% and application of the group II metabotropic glutamate receptor agonist DCGIV (1 µM) at the end of the experiment, which had to block responses completely.

### Drugs

D-(-)-2-Amino-5-phosphonopentanoic acid (D-APV), (2S,2′R,3′R)-2-(2′,3′-Dicarboxycyclopropyl)glycine (DCG IV) and (2S)-2-Amino-2-[(1S,2S9-2-carboxycycloprop-1-yl]-3-(xanth-9-yl)propionic acid (LY341496) were purchased from Tocris International (via Biotrend, Cologne, Germany). All other drugs were obtained from Sigma-Aldrich (Munich, Germany).

## Results

**Figure 1:**
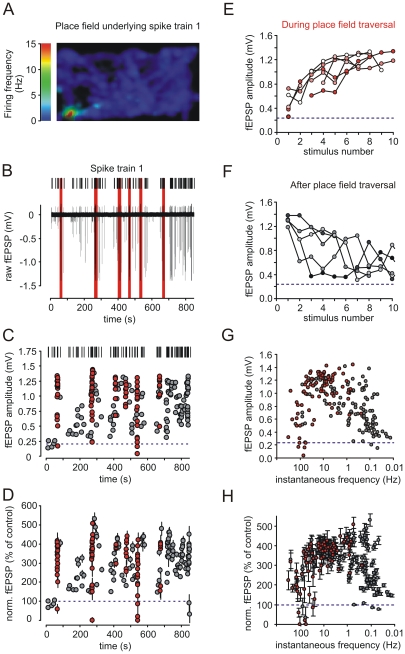
Spiking activity of dentate gyrus granule cells during exploratory behaviour evokes large short-term plasticity at mossy fiber synapses *in vitro*. (**A**) Color-code depicts the location-dependent modulation of firing frequency in Hz of a putative dentate gyrus granule cell while the animal was exploring a rectangular environment. A place field is visible in the down-left corner. (**B**) Time-resolved plot of spike train 1 (up) of a ∼15 minute recording session of putative granule cell activity, that (A) was based on, and continuous recording of mossy fiber fEPSP (down) in response to presentation of that spike train in vitro. Stimulation artifacts were removed for clarity. Red bars indicate episodes inside the place field. (**C**) Representative recording of mossy fiber fEPSPs during response to spike train 1, where highly dynamic fEPSP amplitudes were evoked. Grey indicates responses to spikes outside the cell's place field, red indicates responses within. (**D**) Summary of n = 5 such experiments. Short-term synaptic dynamics were reproducible and comparable between experiments. Data points depict mean ± sem. (**E**) Pronounced short-term facilitation of fEPSPs is induced by stimulus bursts during traversal of place fields. Line graphs show the first 10 mossy fiber fEPSP response amplitudes during six episodes of place field spiking activity (as indicated by red in C). Red circles indicate responses during 1^st^ burst, white circles responses during 6^th^ burst, redish shadings correspond to bursts in between. Single exemplary recording is shown. (**F**) Mossy fiber fEPSP amplitudes decrease again during the first 10 stimuli after place field traversal indicated by grey shadings. Single exemplary recording is shown. (**G/H**) Field EPSP response amplitude as a function of preceding inter-stimulus interval during spike train 1. A large dynamic range of response amplitudes is apparent with amplitudes generally largest with ISIs ranging from 50–1000 ms. (G) depicts single exemplary recording, (H) summarizes n = 6 such experiments. Red circles indicate fEPSP responses during spikes of place field traversal, grey circles such outside of place fields. Blue dashed lines in panels C to H indicate basal response amplitudes to constant stimulation at 0.05 Hz.

We recorded spiking activity of putative hippocampal dentate gyrus granule cells *in vivo* while animals were exploring a rectangular environment. The exemplary granule cell exhibits a single place field in the down-left corner of the rectangular environment. Six visits of this place field could be detected during the shown recording epoch (Figure 1A and B).

We then delivered this 15 minute epoch of continuous *in vivo* granule cell recording (spike train 1) as an extracellular stimulation pattern to hippocampal mossy fibers in an acute slice preparation. Spike train 1 consisted of 223 stimuli with an average frequency of 0.265 Hz, a median inter-stimulus interval (ISI) of 244.4 ms and a total length of 868 seconds. The spike train covered episodes inside and outside of a recognized place field. The corresponding excitatory postsynaptic potentials (fEPSPs) were recorded in stratum lucidum of area CA3. The spike train elicited stable postsynaptic responses, which drastically increased to 400 to 500% of control values during periods of place field activity (n = 5, Figure 1C to E, red circles denote place field responses). After place field traversal the granule cell activity outside of the place field led to gradual decrease of the fEPSP amplitudes to values slightly elevated above pre-place field activity (Figure 1F). This highly dynamic response pattern strongly resembled the frequency facilitation response observed when static stimulation paradigms with a constant frequency of 1 Hz are applied to mossy fibers [9], [11]. During the complete recording epoch the instantaneous granule cell firing frequency ranged from 0.01 to up to 300 Hz. The corresponding postsynaptic response amplitudes were roughly bell-shaped with respect to stimulation frequency and achieved a maximum at frequencies between 1 and 30 Hz (Figure 1G and H), which were mostly reached during place field traversals.

**Figure 2:**
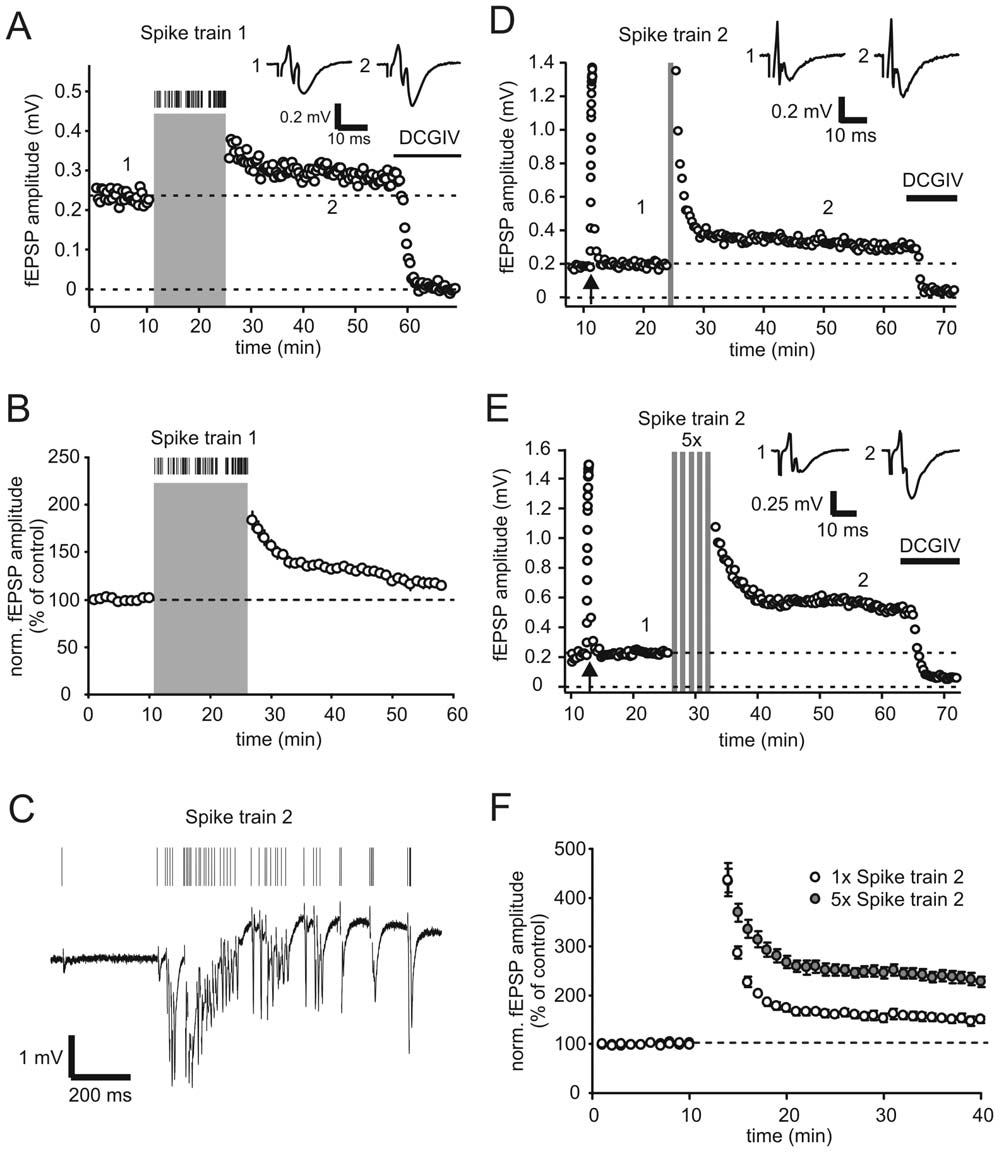
Place field specific spiking activity of dentate gyrus granule cells triggers long-term potentiation of mossy fiber synaptic responses *in vitro*. (**A**) Presentation of spike train 1 (indicated by grey area) led to potentiation of mossy fiber fEPSP amplitudes in this examplary recording. Constant stimulation frequency before and after delivery of spike train was 0.05 Hz. Application of DCGIV at the end of experiment blocked mossy fiber synaptic transmission. Upper traces show averages of 10 sweeps under control condition and 30 min after presentation of spike train 1. (**B**) Summary of n = 5 such experiments. Presentation of spike train led to reliable long-term potentiation of fEPSP amplitudes to ∼130% of control values 25 min after spike train 1. (**C**) Time-resolved plot of another place field specific spike episode (spike train 2, up) and continuous recording of mossy fiber fEPSP response to single presentation of this spike train (lower part). Stimulus artifacts are cut for visual clarity. Please note different timescale compared to spike train 1. (**D**) Examplary mossy fiber synaptic fEPSP recording, where a single presentation of spike train 2 (grey bar, not drawn to scale) leads to long-term potentiation of fEPSP responses. Arrow points to frequency facilitation paradigm (switch of stimulation frequency from 0.05 Hz to 1 Hz for 20 stimuli). Application of DCGIV (1 µM) at the end of experiment blocked mossy fiber fEPSPs. Upper traces show averages of 10 sweeps each under control condition and 25 minutes after presentation of spike train. Constant stimulation frequency was 0.05 Hz. (**E**) Repetitive presentation of spike train 2 (5x with 30 s pauses inbetween) resulted in pronounced long-term potentiation of mossy fiber fEPSP amplitudes in this examplary experiment. Upper traces show averages of 10 sweeps each under control condition and 25 minutes after repetitive presentation of spike train. (**F**) Summary of n = 6 experiments with single presentation of spike train (open circles) and n = 7 experiments with repetitive presentation (filled circles). Both paradigms led to significant long-term potentiation of response amplitudes to ∼150% and ∼230% of control values, respectively. Data shows mean ± sem. Upper dashed lines in subpanels indicate basal response amplitudes to constant stimulation at 0.05 Hz.

To assess any long-lasting effect of spike train 1, we also determined the basal mossy fiber synaptic amplitude in response to a constant stimulation frequency of 0.05 Hz before and after applying spike train 1. Here, we not only found a post-tetanic potentiation within the first minutes after train presentation, but also detected a persistent increase in transmission to 117.5±0.5% of control values as assessed 25 to 30 minutes after the train (n = 5, Figure 2A and B).

In a next set of experiments, we then applied a place field specific spike episode obtained from a different *in vivo* granule cell recording (spike train 2) as published in Henze et al. [17]. Spike train 2 consisted of 45 stimuli with an average frequency of 32.7 Hz, a median inter-stimulus interval (ISI) of 13 ms and a total length of 1.4 seconds (Figure 2C). A single presentation of this short action potential pattern, that contained only place field activity, triggered robust post-tetanic potentiation of over 411±38.2% (n = 7) and to a long-term potentiation of synaptic transmission of 143.7±8.2% (n = 7, Figure 2D and F). Application of 5 repetitions of spike train 1 with 30 second pauses in between, yielded an even stronger potentiation of 227.9±11.3% (n = 7, Figure 2E and F).

**Figure 3:**
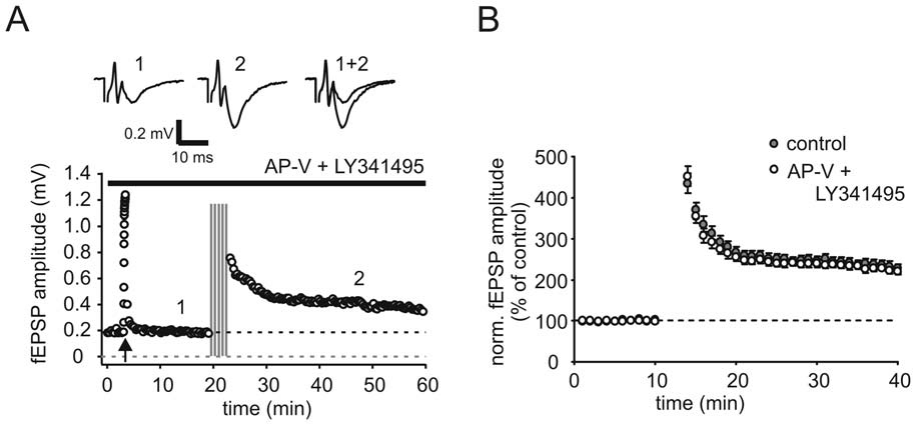
Natural spike trains induce mossy fiber LTP indepedent of NMDAR and mGluR activation. (**A**) Exemplary mossy fiber fEPSP recording under blockage of NMDA- and mGlu-receptors. Repetitive presentation of spike train 2 still induced significant long-term potentiation. Arrow points to frequency facilitation paradigm. Upper traces are averages of 10 sweeps each under control condition and 25 min after presentation of spike trains. Constant stimulation frequency was 0.05 Hz. (**B**) Summary of n = 6 such experiments and experiments under control conditions, respectively. Repetitive presentation of spike train 2 resulted in potentiation of response amplitudes to ∼220% of control values 30 min after presentation of spike trains. Data was binned to 1 min time points and depicts mean ± sem. Upper dashed lines in subpanels indicate basal response amplitudes to constant stimulation at 0.05 Hz.

It is generally agreed upon that induction of classical mossy fiber long-term potentiation (LTP) is independent of NMDA-type ionotropic glutamate receptors, whereas the involvement of group 1 metabotropic glutamate receptors (mGluRs) remains controversial [18], [19]. In order to test for the possible participation of these two receptors in LTP induction with place field associated spiking activity, we again applied spike train 2 in the continuous presence of APV (50 µM) and the broad spectrum antagonist LY341495 (100 µM), to block NMDARs and mGluRs respectively. LTP was readily induced in the presence of the two drugs and was of the same magnitude when compared with control conditions (221.3±8.4%, n = 6, p = 0.66, Figure 3A and B), indicating that here mossy fiber LTP is independent of NMDARs and mGluRs.

**Figure 4:**
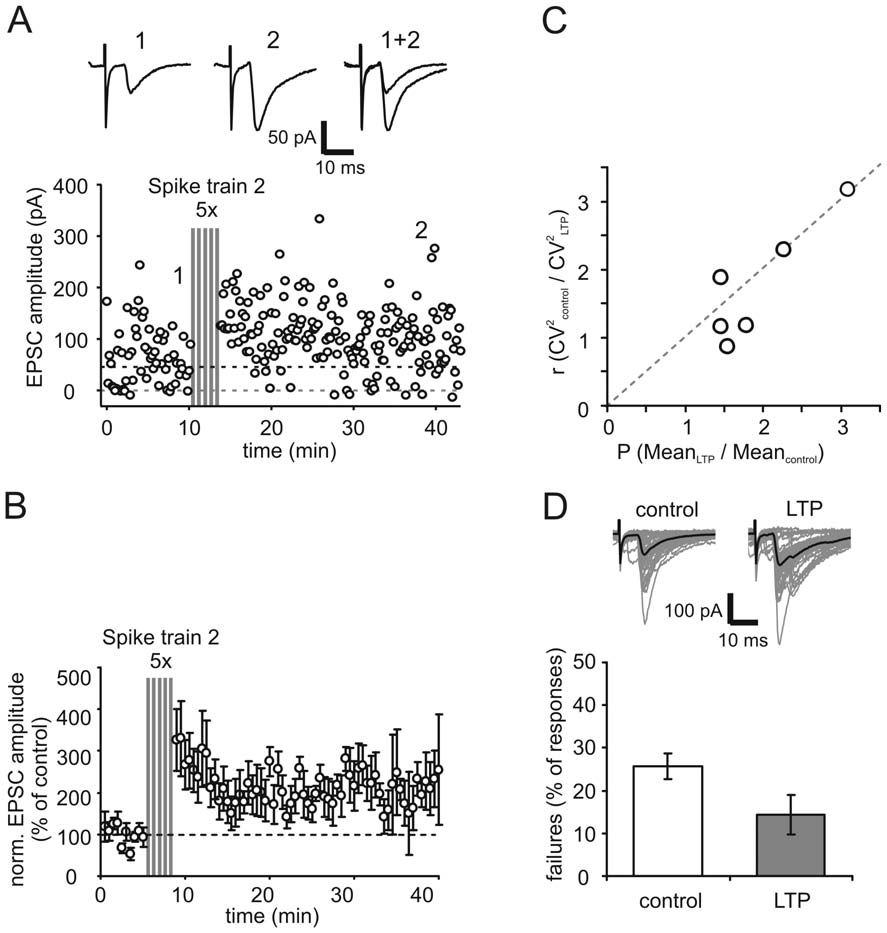
Mossy fiber synaptic LTP - induced by place field specific spiking activity of dentate gyrus granule cells - is presynaptically expressed. (**A**) Examplary whole-cell recording of mossy fiber synaptic responses in CA3 pyramidal cell. Repetitive presentation of spike train 2 (grey bars, compare Figure 2) induces long-term potentiation of EPSC amplitudes. Upper traces show averages of 10 sweeps each under control condition and 20 min after presentation of spike train. Constant stimulation frequency outside of spike train 2 was 0.1 Hz. CA3 pyramidal cell was held in voltage-clamp condition at -60 mV, also during presentation of spike train. Upper dashed line indicates basal response amplitudes to constant stimulation at 0.1 Hz. (**B**) Summary of n = 5 whole-cell experiments where repetitive presentation of spike train 2 induces long-term potentiation of mossy fiber EPSC amplitudes. Potentiation to ∼220% of control values was visible 30 min after spike train. Data was binned to 0.5 min time points and depicts mean ± sem. (**C**) CV^2^ analysis of data from experiments in A. The change in the squared coefficient of variation in control versus LTP condition shows a linear dependence on the change in the mean response amplitude. (**D**) The mean rate of failures of synaptic transmission is decreased after expression of LTP. Upper traces show 50 individual sweeps (grey) and mean sweeps (black) in control and LTP condition of an exemplary whole-cell recording. Note the large incidence of synaptic failures under control conditions.

To investigate the mechanism of long-term potentiation induced by a naturally occurring stimulus train more thoroughly, we performed whole-cell patch-clamp recordings from CA3 pyramidal neurons. In this set of experiments, we used low-intensity extracellular stimulation of presynaptic mossy fibers to test whether single fiber stimulation was sufficient to elicit mossy fiber LTP by natural spike trains. Indeed, five successive applications of spike train 2 induced a robust potentiation of transmission to 220% of control synaptic EPSCs (n = 6, Figure 4A and B). To assure that postsynaptic voltage-dependent Ca^2+^ channels in the CA3 pyramidal cell were not involved in mediating the induction of potentiation, spike train-induced depolarization was blocked during the induction phase by voltage-clamping the postsynaptic cell at −60 mV.

**Figure 5:**
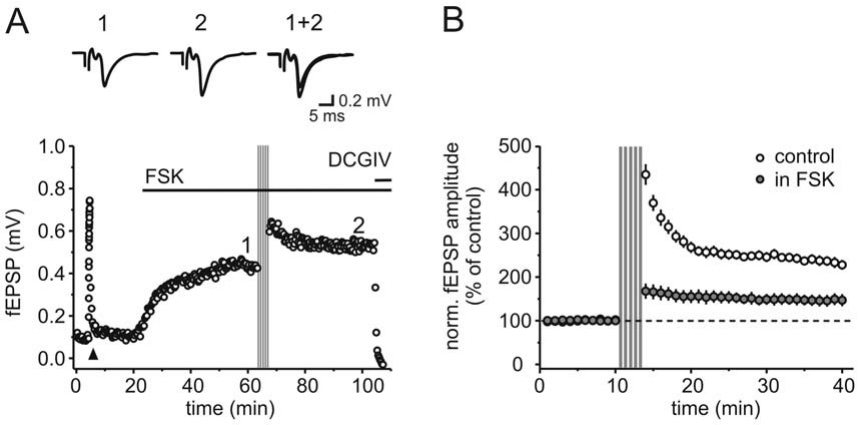
Natural spike train induced long-term potentiation is strongly reduced in the presence of elevated cAMP levels. (**A**) Application of the adenylate cyclase activator forskolin (50 µM) enhances synaptic transmission in this exemplary experiment and strongly reduces long-term potentiation induced by repetitive (5 x) delivery of spike train 2. Traces on top are averages of five consecutive sweeps taken at the time point indicated by the numbers in the graph. Triangle denotes frequency facilitation paradigm for 20 pulses with 1 Hz, arrow indicates time point of spike train 2 application, second horizontal bar represents application of DCGIV (1 µM) at the end of experiment. (**B**) Summary plot displaying the drastically reduced potentiation for n = 4 such experiments (closed circles). Values are normalized to the amplitude in forskolin before train delivery. For comparison, the potentiation elicited by spike train 2 in the absence of drugs (open circles, same dataset as in Figure 2F) is overlayed. In the presence of forskolin the potentiation was reduced to 143.4±11.5% (p<0.001, compared to control).

The expression of classical MF LTP is due to an increase in transmitter release and both pharmacological and genetic analyses indicate that a rise in presynaptic cAMP is a critical component [20], [21]. Thus, we tested whether cAMP-mediated increase in synaptic response amplitude occludes LTP induced by the applied spike trains. Indeed, the application of the adenylate cyclase activator forskolin (50 µM) led to an enhancement of EPSCs amplitudes and reduced spike train induced mossy fiber LTP to 34.3% compared to control values (Figure 5A and B, n = 4).

Further analysis of the synaptic EPSC distributions gained in the minimal stimulation experiments yielded two additional indicators for a presynaptic expression site of long-term potentiation: First, the coefficient of variation before and after induction of LTP by natural spike trains scaled linearly with the response mean (Figure 4C). Second, the use of a minimal stimulation technique in whole-cell recordings enabled us to analyze the amount of transmission failures. We obtained a percentage of 25.7±3.1% of transmission failures under control conditions, similar to [22] where minimal stimulation techniques had been used. During LTP expression, we found the failure rate to be significantly reduced to 14.3±4.7% (Figure 4A and D, n = 6, p<0.05), which is indicative of an increase in synaptic release probability and therefore a presynaptic expression locus.

**Figure 6:**
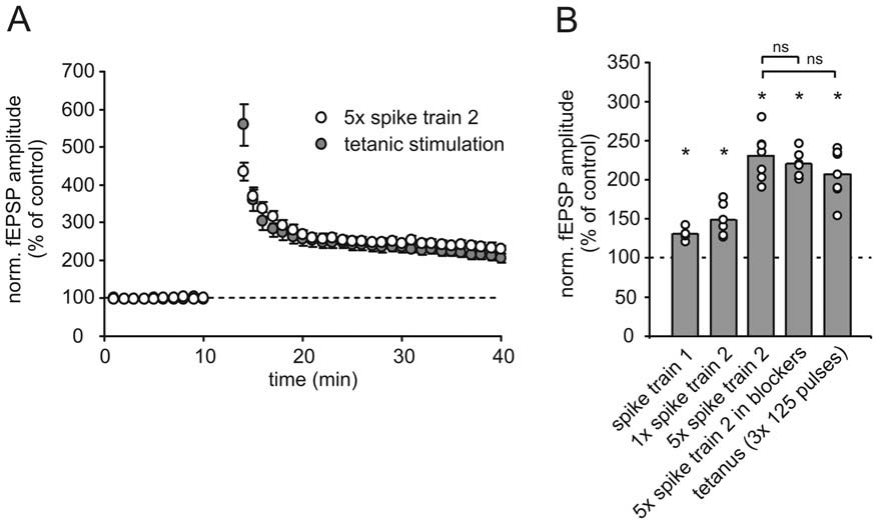
Summary of the amount of potentiation induced by different stimulation paradigms. (**A**) Long-term potentiation induced by repetitive presentation of spike train 2 (open circles) is comparable to LTP induced by a classical LTP induction protocol (grey circles) by tetanic stimulation (3×125 pulses at 25 Hz with 30 s pauses in between). (**B**) All used stimulation paradigms based on place field specific spiking activity of granule cells triggered a significant long-term potentiation of synaptic responses when compared to control values. Asterisks point to a significant increase in fEPSP amplitude (single distribution t-test against 100%). The amount of LTP induced by repetitive presentation of spike train 2 was not significantly (ns) different from LTP induced by a classical tetanic paradigm or under the action of AP-V and LY341496.

In conclusion, we found that mossy fiber synaptic strength is strongly affected on short and long time scales by the presentation of physiological spike trains. Most importantly, different place field associated spike trains effectively trigger long-term potentiation at mossy fiber synapses, which is comparable in amount and mechanism to that of classical static induction paradigms (see Figure 6 for summary).

## Discussion

In general, synapses exhibit activity-dependent plasticity of their transmission efficacy on timescales ranging from milliseconds to days. Classically, these dynamic processes are assessed by constant stimulation paradigms, for example paired-pulse stimuli with varying inter-stimulus intervals to test for short-term plasticity (STP) or high-frequency (tetanic) stimuli to induce long-term plasticity (LTP). While these paradigms are explicitely designed to minimize interfering effects of STP on LTP and are experimentally convenient, they are hardly physiological. More recent studies have focussed on using different physiologically motivated input distributions as stimulation paradigms, representing irregular *in vivo* activity of neurons under various behavioral conditions [23]–[27], which has provided synapse- and systems-specific insights into the quality and amount of plasticity.

### Characteristics of natural spike trains

Spiking activity of hippocampal principle cells varies with the location of behaving rodents, with firing frequencies rapidly increasing to values up to 20 to 30 Hz while the animal traverses a so-called place field [3]. The characteristics, formation and modifiability of such place cell firing CA1 and CA3 pyramidal neurons have been investigated in detail [28] [29] [30]. To date, descriptions of typical dentate gyrus granule cell firing activity under in vivo conditions are, however, sparse due to difficulties in effective spike sorting and cell assignement. Granule cells do also exhibit place field firing, with short, high-frequent spike episodes inside a place field and episodes of low firing outside, but the published data on mean values and ranges of spike frequencies as well as inter-spike interval distributions vary significantly between studies and behavioral conditions. Mean firing rates range from 0.2 Hz during locomotor behavior [7], to up to behaviorally coupled 10 Hz [31], to a mean rate of 1.7 Hz outside or 26 Hz inside a place field during freely behaving locomotion [17]. In our study, we have used several complementary examples of dentate gyrus granule cell spiking activity during exploratory behavior, covering episodes inside and outside of detected place fields. We therefore believe that our results reflect a representative description of short- and long-term plasticity events at the mossy fiber synapse occuring under spiking activity as found *in vivo*.

### Short-term plasticity induced by natural spike trains at mossy fiber synapses

The hippocampal mossy fiber synapse is well known to exhibit remarkable short-term plasticity with paired-pulse ratios of up to 4 and frequency facilitation of synaptic amplitudes under 1 Hz stimulation to at least 400%. [9] [11]. An exceptionally large range of postsynaptic response amplitudes is also observed with irregular presynaptic stimulation [12]. In our experiments, we observed comparable increases in synaptic responses of up to 500% when mossy fibers were stimulated with patterns of naturally occuring granule cell activity. Large facilitation was prominent during place-field related high-frequent activity, while synaptic responses slowly decreased again during low-frequent episodes between place-field crossings. The postsynaptic responses increase proportionally with higher instantaneous stimulation frequencies with a maximum of facilitation at 10 to 30 Hz. Responses facilitated slightly less at even higher stimulation frequencies between 30 and 300 Hz. This predominantly facilitatory short-term plasticity is prone to generate high-pass filtering characteristics of the synapse, enabling high reliability of transmission during burst-like activity epochs [26], [32], [33]. The strong facilitation and large dynamic range of postsynaptic responses clearly separates the mossy fiber synapse from other synaptic systems [23], [24], [26]. In particular, the hippocampal Schaffer collateral to CA1 synapses have been described to exhibit a bandpass of even switch-like filtering behavior when tested with natural input statistics [26]. In general, the short-term dynamics reported here were highly reproducible in recordings from different slices and thus give a representative description of mossy fiber synaptic short-term plasticity in response to natural input statistics.

### Long-term synaptic plasticity induced by natural spike activity

In addition to eliciting prominent short-term dynamics, the delivery of place-field associated spike trains on a longer timescale led to robust potentiation of mossy fiber synaptic responses. Both spike train 1 and spike train 2, in single and multiple applications, triggered a significant and long-lasting enhancement of transmission, which was comparable to the amount of potentiation typically described when using classical LTP induction paradigms.

Regarding the mechanism of induction, there is rather unequivocal agreement that classical mossy fiber LTP is NMDAR independent, while some controversy exists about the necessity of mGluR activation [11]. In this context, strong induction protocols [18] or a newly discovered NMDAR dependent postsynaptic form of mossy fiber LTP [34] [35] have been shown to rely on mGluR5 activation. In the experiments presented here, we found the induction of LTP through natural spike trains to be independent of NMDAR and mGluR activation, as LTP could readily be induced in the presence of the respective receptor antagonists.

The mechanism of expression of classical mossy fiber LTP has been shown to be presynaptically located and based on an increase in the probability of transmitter release (Pr) [11]. In this study, several lines of evidence suggest that mossy fiber long-term potentiation induced by natural spike trains likewise leads to a presynaptic inrease in Pr: (1) In whole-cell recordings of CA3 pyramidal neurons using a minimal stimulation procedure the number of transmission failures decreased after LTP induction with spike train 2, (2) the analysis of the coefficient of variation shows that the CV2 is scaled linearly with the response amplitude after LTP induction [36], and finally, (3) the paired-pulse ratio decreased after LTP.

### Effects of natural spiking activity on network function

Several previous *in vitro* studies focussing on the CA1 region of the hippocampus have already highlighted the importance of using natural input statistics to fully unravel the characteristics of synaptic dynamics. Place-field associated spike trains trigger short-term plasticity at Schaffer collateral to CA1 pyramidal cell synapses composed of an overlap of facilitation and depression, while static stimulation paradigms yield either faciliation or depression depending on the developmental stage [24]. Application of high-frequency episodes of place cell firing via bulk stimulation also induces LTP at Schaffer collateral to CA1 pyramidal neurons [23], while the induction of LTP in CA1 pyramidal cells on a single-cell level appears to require temporally coordinated pre- and postsynaptic firing sequences from overlapping place fields and the correct level of cholinergic neuromodulatory tone [27]. Such results illustrate the short- and long-term impacts of even very brief natural spike trains on synaptic functioning, which consequently should also alter the computational principles of the underlying network. These findings are well reflected in recent *in vivo* work, where recordings of the large-scale network activity in the CA1 area of the hippocampus revealed very distinct spiking ensembles under control behavior and during startling conditioning paradigms as well as immediate encoding of associative memory traces [37] [38]. Appropriate pre- and postsynaptic firing statistics are certainly required to induce such large functional network rearrangements and these should manifest themselves also at the synaptic level.

Summarizing our results, we here could show that physiological granule cell spiking activity is sufficient and effective in triggering both synaptic short-term dynamics of large range and a presynaptically induced and expressed form of mossy fiber long-term potentiation. These findings underline the importance to further elucidate the detailed signalling cascades involved in this peculiar form of plasticity at hippocampal mossy fiber synapses – as it does indeed occur with natural spiking activity.

## References

[1] Malenka RC, Nicoll RA (1999). Long-term potentiation–a decade of progress?. Science.

[2] Zucker RS, Regehr WG (2002). Short-term synaptic plasticity.. Annu Rev Physiol.

[3] O'Keefe J (1979). A review of the hippocampal place cells.. Prog Neurobiol.

[4] O'Keefe J, Dostrovsky J (1971). The hippocampus as a spatial map. Preliminary evidence from unit activity in the freely-moving rat.. Brain Res.

[5] Wilson MA, McNaughton BL (1993). Dynamics of the hippocampal ensemble code for space.. Science.

[6] Leutgeb JK, Leutgeb S, Moser MB, Moser EI (2007). Pattern separation in the dentate gyrus and CA3 of the hippocampus.. Science.

[7] Jung MW, McNaughton BL (1993). Spatial selectivity of unit activity in the hippocampal granular layer.. Hippocampus.

[8] Buzsaki G, Czeh G (1992). Physiological function of granule cells: a hypothesis.. Epilepsy Res.

[9] Salin PA, Scanziani M, Malenka RC, Nicoll RA (1996). Distinct short-term plasticity at two excitatory synapses in the hippocampus.. Proc Natl Acad Sci U S A.

[10] Henze DA, Urban NN, Barrionuevo G (2000). The multifarious hippocampal mossy fiber pathway: a review.. Neuroscience.

[11] Nicoll RA, Schmitz D (2005). Synaptic plasticity at hippocampal mossy fibre synapses.. Nat Rev Neurosci.

[12] Gundlfinger A, Leibold C, Gebert K, Moisel M, Schmitz D (2007). Differential modulation of short-term synaptic dynamics by long-term potentiation at mouse hippocampal mossy fibre synapses.. J Physiol.

[13] Mizumori SJ, McNaughton BL, Barnes CA (1989). A comparison of supramammillary and medial septal influences on hippocampal field potentials and single-unit activity.. J Neurophysiol.

[14] Harris KD, Henze DA, Csicsvari J, Hirase H, Buzsaki G (2000). Accuracy of tetrode spike separation as determined by simultaneous intracellular and extracellular measurements.. J Neurophysiol.

[15] Hazan L, Zugaro M, Buzsaki G (2006). Klusters, NeuroScope, NDManager: a free software suite for neurophysiological data processing and visualization.. J Neurosci Methods.

[16] Schmitz D, Mellor J, Breustedt J, Nicoll RA (2003). Presynaptic kainate receptors impart an associative property to hippocampal mossy fiber long-term potentiation.. Nat Neurosci.

[17] Henze DA, Wittner L, Buzsaki G (2002). Single granule cells reliably discharge targets in the hippocampal CA3 network in vivo.. Nat Neurosci.

[18] Yeckel MF, Kapur A, Johnston D (1999). Multiple forms of LTP in hippocampal CA3 neurons use a common postsynaptic mechanism.. Nat Neurosci.

[19] Mellor J, Nicoll RA (2001). Hippocampal mossy fiber LTP is independent of postsynaptic calcium.. Nat Neurosci.

[20] Huang YY, Li XC, Kandel ER (1994). cAMP contributes to mossy fiber LTP by initiating both a covalently mediated early phase and macromolecular synthesis-dependent late phase.. Cell.

[21] Weisskopf MG, Castillo PE, Zalutsky RA, Nicoll RA (1994). Mediation of hippocampal mossy fiber long-term potentiation by cyclic AMP.. Science.

[22] Mori-Kawakami F, Kobayashi K, Takahashi T (2003). Developmental decrease in synaptic facilitation at the mouse hippocampal mossy fibre synapse.. J Physiol.

[23] Dobrunz LE, Stevens CF (1999). Response of hippocampal synapses to natural stimulation patterns.. Neuron.

[24] Dekay JG, Chang TC, Mills N, Speed HE, Dobrunz LE (2006). Responses of excitatory hippocampal synapses to natural stimulus patterns reveal a decrease in short-term facilitation and increase in short-term depression during postnatal development.. Hippocampus.

[25] Frerking M, Ohliger-Frerking P (2006). Functional consequences of presynaptic inhibition during behaviorally relevant activity.. J Neurophysiol.

[26] Klyachko VA, Stevens CF (2006). Excitatory and feed-forward inhibitory hippocampal synapses work synergistically as an adaptive filter of natural spike trains.. PLoS Biol.

[27] Isaac JT, Buchanan KA, Muller RU, Mellor JR (2009). Hippocampal place cell firing patterns can induce long-term synaptic plasticity in vitro.. J Neurosci.

[28] Leutgeb S, Leutgeb JK, Treves A, Moser MB, Moser EI (2004). Distinct ensemble codes in hippocampal areas CA3 and CA1.. Science.

[29] O'Keefe J, Burgess N (1996). Geometric determinants of the place fields of hippocampal neurons.. Nature.

[30] Lee I, Yoganarasimha D, Rao G, Knierim JJ (2004). Comparison of population coherence of place cells in hippocampal subfields CA1 and CA3.. Nature.

[31] Wiebe SP, Staubli UV (1999). Dynamic filtering of recognition memory codes in the hippocampus.. J Neurosci.

[32] Lisman JE (1997). Bursts as a unit of neural information: making unreliable synapses reliable.. Trends Neurosci.

[33] Kepecs A, Lisman J (2003). Information encoding and computation with spikes and bursts.. Network.

[34] Kwon HB, Castillo PE (2008). Long-term potentiation selectively expressed by NMDA receptors at hippocampal mossy fiber synapses.. Neuron.

[35] Rebola N, Lujan R, Cunha RA, Mulle C (2008). Adenosine A2A receptors are essential for long-term potentiation of NMDA-EPSCs at hippocampal mossy fiber synapses.. Neuron.

[36] Faber DS, Korn H (1991). Applicability of the coefficient of variation method for analyzing synaptic plasticity.. Biophys J.

[37] Lin L, Osan R, Shoham S, Jin W, Zuo W (2005). Identification of network-level coding units for real-time representation of episodic experiences in the hippocampus.. Proc Natl Acad Sci U S A.

[38] Chen G, Wang LP, Tsien JZ (2009). Neural population-level memory traces in the mouse hippocampus.. PLoS One.

